# Evaluating tools for transcription factor binding site prediction

**DOI:** 10.1186/s12859-016-1298-9

**Published:** 2016-11-02

**Authors:** Narayan Jayaram, Daniel Usvyat, Andrew C. R. Martin

**Affiliations:** Institute of Structural and Molecular Biology, Division of Biosciences, University College London, Darwin Building, Gower Street, London, WC1E 6BT UK

**Keywords:** PWMs, Motif discovery, Performance evaluation, Motif scanning tools

## Abstract

**Background:**

Binding of transcription factors to transcription factor binding sites (TFBSs) is key to the mediation of transcriptional regulation. Information on experimentally validated functional TFBSs is limited and consequently there is a need for accurate prediction of TFBSs for gene annotation and in applications such as evaluating the effects of single nucleotide variations in causing disease. TFBSs are generally recognized by scanning a position weight matrix (PWM) against DNA using one of a number of available computer programs. Thus we set out to evaluate the best tools that can be used locally (and are therefore suitable for large-scale analyses) for creating PWMs from high-throughput ChIP-Seq data and for scanning them against DNA.

**Results:**

We evaluated a set of *de novo* motif discovery tools that could be downloaded and installed locally using ENCODE-ChIP-Seq data and showed that rGADEM was the best-performing tool. TFBS prediction tools used to scan PWMs against DNA fall into two classes — those that predict individual TFBSs and those that identify clusters. Our evaluation showed that FIMO and MCAST performed best respectively.

**Conclusions:**

Selection of the best-performing tools for generating PWMs from ChIP-Seq data and for scanning PWMs against DNA has the potential to improve prediction of precise transcription factor binding sites within regions identified by ChIP-Seq experiments for gene finding, understanding regulation and in evaluating the effects of single nucleotide variations in causing disease.

**Electronic supplementary material:**

The online version of this article (doi:10.1186/s12859-016-1298-9) contains supplementary material, which is available to authorized users.

## Background

The sequence-specific binding of transcription factors to transcription factor binding sites (TFBSs) is key to the mediation of transcriptional regulation [1]. High throughput experimental methods for identifying TFBSs such as ChIP-Chip and ChIP-Seq identify a region of 100–1000 base pairs (b.p.) while the actual TFBS is a short region (typically 9–15 b.p.) within that region. Nonetheless, there is a small set of experimentally precisely validated functional transcription factor binding sites which are stored in reference databases such as PAZAR [2] and ORegAnno [3]. However this is an insignificant proportion of transcription factor binding sites in terms of the human genome. Hence there is a need for accurate computational prediction of transcription factor binding sites [4], for gene finding, understanding regulation and in applications such as evaluating the effects of single nucleotide variations (SNVs) in causing differential expression [4] and leading to disease [5].

Prediction of transcription factor binding sites is generally performed by scanning a DNA sequence of interest with a position weight matrix (PWM) for a transcription factor of interest [6, 7] and various pattern-matching tools have been developed for this purpose. These tools fall into two classes: those that predict clusters of transcription factor binding sites or those that predict individual sites.

### Experimental identification of transcription factor binding sites

There are many in vitro and in vivo experimental approaches that have been used to identify transcription factor binding sites and these are reviewed briefly here.

In vitro methods include: (i) The **Electro-Mobility Shift Assay (EMSA)** [8] which exploits the ability of a non-denaturing polyacrylamide gel to act as a molecular sieve, separating protein-bound DNA from unbound DNA. (ii) The **DNase I footprinting/protection** assay combines the cleavage reaction of DNase I with EMSA [9]. A key problem with both EMSA and DNase I footprinting is the identification of unwanted protein-DNA interactions that result from non-specific DNA binding proteins [8]. (iii) **Systematic Evolution of Ligands by EXponential enrichment (SELEX)** [10] works by screening a large pool of short, random oligonucleotide probes which are recognized by a TFBS of interest [10]. A refinement, SELEX-seq, involves the selected dsDNAs being subjected to massively parallel sequencing [11].

There has been a recent shift towards in vivo approaches [4]. In the (iv) **Chromatin ImmunoPrecipitation (ChIP)** assay, a variation of the ‘pull down’ class of assay [12], the DNA-binding protein of interest is cross-linked to the DNA using formaldehyde. The DNA is then fragmented into small fragments of around 100–1000 b.p. and an antibody specific for a given transcription factor is then used to immunoprecipitate the DNA-protein complex. The cross-links are then reversed, releasing the DNA for PCR amplification [12]. High throughput versions of the ChIP assay involve hybridizing the resulting fragments to genomic tiling microarrays, an approach known as ChIP-chip [13], or the resulting DNA fragments can undergo massively parallel sequencing, an approach known as ChIP-Seq [14].

There are a number of advantages of using ChIP-Seq instead of ChIP-chip. Key improvements are in base pair resolution, avoiding non-linearity and saturation of ChIP-chip signal intensity, ability to analyze sequence repeat regions, and avoiding limitations from the limited selection of probes on a ChIP-chip array. Overall ChIP-Seq has a higher specificity and sensitivity compared with ChIP-chip [14, 15] and has largely superseded the ChIP-chip method. Consequently, ChIP-Seq is the current ‘gold standard’ for identifying protein/DNA interactions sites such as histone modifications as well as transcription factor binding sites [16]. A recent refinement to ChIP-Seq is ChIP-exo where the resulting fragments from the ChIP assay are trimmed using lambda exonuclease. This results in fragments that are shorter (∼50 b.p.), but still larger than the precise TFBS [17].

### Position weight matrices (PWMs)

Position Weight Matrices (PWMs) are the most widely used approach to modelling TFBSs. In contrast to a consensus model (which simply gives the most common base(s) at each position of a binding motif), a matrix-based PWM model (which is simply a 4×*n* matrix of scores for each of the 4 bases across each position in the binding motif) accounts for the preference for each of the four nucleotides at each position in the motif [4, 6, 18].

The high-throughput techniques, particularly ChIP-Seq and SELEX-seq, provide an opportunity to identify and characterize protein-DNA binding events at a genome-wide level, contrary to the previous techniques that were only able to characterize a small number of protein-DNA binding events. Hu et al. [19] have suggested that PWMs derived from transcription factor binding sites detected by these methods will be more accurate than PWMs derived from techniques such as SELEX, or compilations of individual promoter assays that detect limited transcription factor binding site numbers. Further, the ChIP-Seq technique has been found to produce PWMs with greater accuracy than ChIP-chip owing to the superior resolution provided by the ChIP-Seq technique [19, 20].

PWMs can be obtained from a number of resources including the commercial database TRANSFAC [21] and the open access database JASPAR [20]. TRANSFAC PWMs are derived from experimental evidence obtained from the literature [21], but availability and application is limited by a commercial licence. The bulk of the PWMs in earlier versions of JASPAR were derived from SELEX experiments and individual promoter assays, but since 2014, updates to JASPAR [22] now include new PWMs derived from ChIP-Seq data using MEME for motif discovery. Other recent resources include HOCOMOCO [23], HOMER ([24] http://homer.salk.edu/homer/motif/HomerMotifDB/homerResults.html) and CIS-BP [25]. However, JASPAR is a well-established and widely-used resource that was employed by us in previous unpublished work and consequently was used in some of the work presented here.

### *de novo* motif discovery

While large scale ChIP experiments allow the genome-wide identification of binding regions for a specific transcription factor, these regions are much longer than the actual binding site for a specific transcription factor meaning that the actual transcription factor binding sites still need to be identified [26, 27].

Various motif discovery methods have been developed and there have been several reviews of the approaches used ([28–34], for example). The most popular algorithms are either enumerative or probabilistic. Enumerative methods examine frequencies of all DNA strings forming a PWM from the over-represented strings that have been identified [1]. Probabilistic methods generate a local multiple alignment of sequences while learning the parameters of the PWM using approaches such as expectation-maximization [35], Gibbs sampling [36], or greedy approaches [37]. The advantage of enumerative methods is that there is less chance of them getting stuck in a local optimum, while probabilistic methods can cope with arbitrary motif model variations and hence remain unaffected by motif length [1]. For example, the well-known motif discovery program MEME [38] uses a probabilistic method with expectation-maximization [39].

*De novo* motif discovery has proved to be challenging when carried out on the binding regions resulting from the genome-wide techniques of ChIP-chip and ChIP-Seq using conventional motif discovery programs such as MEME, owing to the large volumes of data generated by these techniques; ChIP-Seq can generate over 10,000 sequences in a single run. Hence a common practice has been to use these tools on a subset of the sequences [19, 40, 41]. However, Hu et al. [19] have suggested that this practice will lead to inaccurate PWMs and consequently new tools have recently been developed that are able to handle the large volumes of data generated from these high-throughput technologies. These include the freely available software packages ChIPMunk [42], HOMER (Hypergeometric Optimization of Motif EnRichment) [24], rGADEM (Genetic Algorithm guided formation of spaced Dyads coupled with EM for Motif identification) [43] and MEME-ChIP [44, 45].

### Evaluation of the performance of transcription factor binding site prediction tools and motif discovery tools

As well as high quality PWMs to model TFBSs, the computational prediction of TFBSs requires a pattern matching tool. A number of tools are available for this purpose which fall into two classes: those that predict clusters of sites and those that predict individual sites. Consequently the range of tools that can be used locally for motif discovery designed for use with high-throughput data and tools for identifying TFBSs using PWMs warrants an independent performance evaluation.

Approaches for scanning PWMs against DNA were reviewed by Hannenhalli [6] and by Bulyk [30], but the number of performance comparisons is limited. Most have been as part of authors’ evaluations of their own new tools ([46, 47], for example) although an independent assessment was performed by Roulet et al. [48] and a much more recent survey of online PWM scanning tools was performed by Tran and Huang [49].

A number of authors have performed comparisons of methods for motif discovery. These include work by Sandve and colleagues [32, 50, 51], McLeay and Bailey [52] and Orenstein et al. [53]. Kibet and Machanick [34] assessed the performance of matrices obtained from different sources, but did not directly assess the motif discovery tools. The most comprehensive evaluations of tools are those performed by Tompa et al. [39], Hu et al. [54], Medina-Rivera et al. [55] and, most recently, Weirauch et al. [56]. Tompa et al. [39] performed an independent assessment of the performance of 13 tools designed for discovery of novel regulatory elements with no a priori knowledge of the transcription factor involved. They made predictions across a number of species (fly, human, mouse and yeast) with known binding sites taken from TRANSFAC. Assessment was performed at a nucleotide level (i.e. whether individual bases were correctly identified as being part of a binding motif or not) and they concluded that, overall, Weeder [57] performed best. Hu et al. [54] performed another assessment around the same time. However, while Tompa et al. allowed the authors of tools to fine-tune parameters to achieve what they considered to be the best results, Hu et al. performed minimum intervention reflecting the approach likely to be taken by the average end user. They assessed five methods at different levels: nucleotide, binding site, sequence and motif. They also created a ‘consensus ensemble algorithm’ which exploits variations in predictions by stochastic methods to refine predictions. Neither Tompa et al. nor Hu et al. assessed the quality of any models (PWMs) generated from these motifs by applying them to search for TFBSs in DNA.

More recently, Kibet and Machanick [34] reviewed and evaluated different approaches and pointed out the difficulty in evaluating motif discovery tools by applying the PWMs to motif searching: annotation of precise true TFBSs in DNA, to use as a gold standard reference set, is limited. An assessment of motif discovery methods using binding site prediction for evaluation was performed by Medina-Rivera et al. [55]. They generated an assessment method that combines theoretical and empirical score distributions to assess reliability of PWMs for predicting TFBSs and used this to analyze PWMs for bacterial, yeast and mouse TFBSs. Weirauch et al. [56] evaluated 26 tools for motif discovery using in vitro data for 66 mouse TFBSs, looking at PWMs and more complex models such as dinucleotide matrices and secondary motifs. They added ChIPMunk [42] and MEME-ChIP [44, 45] for a further evaluation of performance on in vivo data using five mouse and four yeast TFBSs. During this comparison they found that ChIPMunk outperforms MEME-ChIP.

In this paper we conduct an independent assessment of a set of four motif discovery tools specifically designed for handling large datasets from high-throughput methods (including ChIPMunk and MEME-ChIP evaluated by Weirauch et al.), but using human ChIP-Seq data obtained from ENCODE [58]. Performance evaluation makes use of a gold standard reference set of experimentally-validated precise human transcription factor binding sites. We also evaluate a number of open source PWM scanning tools that are well documented and can be installed locally and are therefore more suitable for large scale analyses. These pattern matching tools represent both classes (individual and cluster).

## Methods

### Sources of experimentally validated TFBSs

To evaluate performance, we identified experimentally-validated TFBSs from resources that, rather than just providing PWMs or approximate regions to which TFBSs bind, provide precise validated binding sites for a limited set of genes. Three sources of such data are available: PAZAR [2], ORegAnno [3, 59] and TRANSFAC [21]. TRANSFAC was rejected because of its commercial licensing, while the data in PAZAR are a superset of ORegAnno and consequently, the PAZAR dataset was selected.

### Selecting data from PAZAR

PAZAR contains some redundancy (multiple instances of the same TFBS annotated for a given gene), so any duplicate TFBSs were removed.

**Fig. 1:**
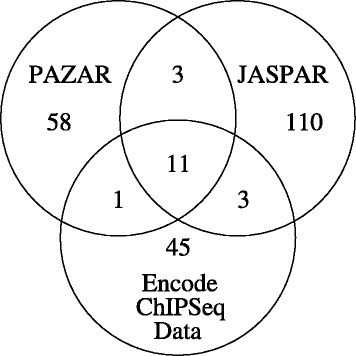
Overlap of transcription factor data. The Venn diagram shows overlaps between known sites in PAZAR, the PWMs in JASPAR and those derived from the ENCODE-ChIP-Seq data used in this paper

PAZAR contains 159 genes annotated with TFBSs that are contained in either JASPAR or ENCODE-ChIP-Seq data. This set contains data for 14 TFBSs with corresponding PWMs in JASPAR coming from a total of 156 human genes. This set is referred to below as ‘PAZAR-J’. The set also contains data for 12 transcription factors with binding data in the ENCODE-ChIP-Seq data which come from a total of 149 genes (‘PAZAR-E’). The PAZAR-J and PAZAR-E datasets overlap for 11 of the transcription factors (See Fig. 1 and Additional file 1 for details.)

### Tool evaluation

Initial evaluation of the motif scanning tools (using PWMs from the 2010 release of JASPAR that we had used in earlier work, referred to here as JASPAR.2010) was performed for each of the 14 transcription factors in PAZAR-J by selecting the appropriate subset of the 156 genes in PAZAR-J having validated binding sites for the transcription factor in question.

Evaluation of the motif discovery tools was performed for each of the 12 transcription factors in PAZAR-E by selecting the appropriate subset of the 149 genes in PAZAR-E having validated binding sites for the transcription factor in question and using the motif discovery tool selected in the initial evaluation (FIMO).

Finally, re-evaluation of the motif scanning tools (using PWMs generated by rGADEM) was also performed for each of the 12 transcription factors in PAZAR-E by selecting the appropriate subset of the 149 genes in PAZAR-E having validated binding sites for the transcription factor in question.

### DNA Data

TFBSs can occur in the promoter region, in introns and exons, and far upstream of genes [60, 61]. Consequently the complete gene sequence (i.e. both exons and introns), together with an upstream region of 10,000 b.p. of each of the genes was obtained from Biomart [62] using the biomaRt package in Bioconductor [63–65].

### Performance Metrics

True positives (*TP*) were defined as predicted binding sites having a minimum overlap of 70 % of base pairs with known binding sites from PAZAR. Similarly, false positives (*FP*) were defined as predicted binding sites not having an overlap of at least 70 % with a known binding site and false negatives (*FN*) were defined as known binding sites that were not identified. Obtaining a true estimate of the total number of negative sites (and hence the number of true negatives, *TN*) is difficult and therefore we adopted the normal practice of avoiding performance measures that require true negative counts [66]. For cluster predictors, *all* predicted component TFBSs within a region must overlap with known sites by at least 70 % of base pairs for a prediction to be regarded as a true positive.

As a control, all the DNA sequences were scrambled using the ‘shuffleseq’ program from the EMBOSS suite (version 6.4.0) [67]. In this case there are no actual positives and therefore no true positives or false negatives. Any positive predictions are therefore classified as false positives and the number of actual negatives (*AN*=*FP*+*TN*) was defined as *AN*=*L*/*l*_*t*_ where *L* is the length of the sequence and *l*_*t*_ is the length of the PWM in question).

Performance was assessed by calculating sensitivity (*Sn*=*TP*/(*TP*+*FN*)), positive predictive value (*PPV*=*TP*/(*TP*+*FP*)) and geometric accuracy ($ {ACC}_{g} = \sqrt { {Sn}. {PPV}}$) [66], averaged across the TFBS PWMs and genes analyzed. For the scrambled sequences, a false positive rate was calculated (*FPR*_*s*_=*N*_*p*_/*AN*, where *N*_*p*_ is the number of predicted sites and *AN* is the number of actual negatives as defined above).

### Derivation of PWMs

**Fig. 2:**
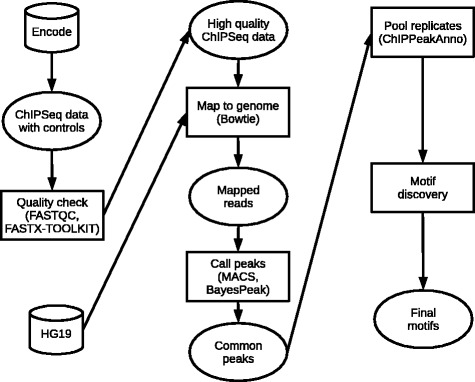
Flowchart summarising the methods used to derive PWMs from the ENCODE-ChIP-Seq data. See text for details

The methods used for deriving PWMs from the ENCODE-ChIP-Seq data are summarized in Fig. 2.

ChIP-Seq data for the human transcription factors were obtained from the ENCODE project (http://hgdownload.cse.ucsc.edu/goldenPath/hg19/encodeDCC/wgEncodeSydhTfbs/) in FASTQ format. Only the ChIP-Seq data that had a corresponding control sample available were selected to help to control biases and artefacts that occur in the experimental protocol [14, 68]. ChIP-Seq control samples are obtained from a mock experiment without the specific antibody and were used during the peak calling process as recommended by Bardet et al. [68]. It is critical that the short reads arising from ChIP-Seq are aligned properly to the reference genome, otherwise false positives and false negatives would occur. Thus, low quality reads and adaptor sequences were identified using FASTQC (http://www.bioinformatics.babraham.ac.uk/projects/fastqc/) and removed using the FASTX TOOLKIT (http://hannonlab.cshl.edu/fastx_toolkit/).

The reads were then mapped to the human genome version hg19 using Bowtie [69]. The resulting Sequence Alignment/Map format (SAM) files were converted to binary format (BAM) files and indexed using SAMtools [70]. This step reduces the file size and allows rapid access which is essential given the large size of the data.

After the reads were aligned to the reference genome, peak calling was performed by identifying statistically significant binding regions that are enriched in the ChIP-Seq sample compared with the control sample [14]. It has been suggested that peaks should be called using more than one peak caller and the intersection of peaks should then be taken [71]. Consequently peaks were called using both MACS [72] and the bioconductor package BayesPeak [63, 73, 74]. Common peaks were identified and replicates were pooled using the bioconductor package ChIPpeakAnno [63, 75]. A set of peak regions — centred on the summits of the peaks (±100 b.p.) in order to prevent bias towards longer peak regions [68] — were obtained in FASTA format. We refer to these filtered peak data as the ‘ENCODE-ChIP-Seq data’.

The TFBS motif discovery tools evaluated were MEME-ChIP [44, 45], HOMER [24], ChIPMunk [42] and rGADEM [43, 63] and these were tested using the 12 transcription factors in the PAZAR-E dataset. Since these programs are able to deal with large datasets, all peak regions were used. The motif discovery methods have various adjustable parameters and these were explored in 10 % steps.

## Results and discussion

As shown in Fig. 1, the overlap between transcription factors having validated binding sites in PAZAR, the PWMs describing these TFBSs in JASPAR and the binding sites in the ENCODE-ChIP-Seq data is fairly small. Only 11 transcription factors (E2F1, ELK4, GATA2, GATA3, IRF1, MAX, NF- *κ*B, STAT1, YY1, CTCF and NFYA) have validated TFBSs in PAZAR, PWMs in JASPAR and are also represented in the ENCODE-ChIP-Seq data. BRCA1 was also found in all datasets, but has recently been removed from JASPAR since its sequence specificity has been questioned [76]. While the ENCODE-ChIP-Seq data are actually more comprehensive than indicated, only ChIP-Seq datasets having no access restrictions and for which the transcription factor had a control ChIP-Seq sample available were chosen. Three additional transcription factors have data that overlap between JASPAR and the ENCODE-ChIP-Seq data (USF2, ZNF263 and JUND) and between PAZAR and JASPAR (ESR1, ESR2 and SP1) while one more has data that overlaps between PAZAR and the ENCODE-ChIP-Seq data (TAL1). Consequently the number of PWMs that could be used for the evaluations described below was limited to 11–14.

Logically it makes sense to evaluate motif discovery methods first and then to evaluate the tools available for matching the derived PWMs to DNA sequences. However the evaluation of the performance of motif discovery methods requires a tool to test the performance of the resulting PWMs. Therefore we needed to select a motif scanning tool for this purpose. In earlier work we had tested the performance of a number of PWM scanning tools using older JASPAR matrices (JASPAR.2010). These results are summarized below and the best performing tool was then used for evaluating the motif discovery methods. Finally, the performance of the scanning tools was reassessed using motifs from the best performing motif discovery method.

### Selecting a PWM scanning tool for evaluation of motif discovery methods

As stated above, in order to evaluate motif discovery methods, we need to scan the motifs against DNA and compare the predictions with a gold-standard set of known precise TFBSs. In work done in 2011, we evaluated the performance of different PWM scanning tools using the older JASPAR.2010 matrices [20] which had been derived from SELEX and individual promoter assays. Consequently, we exploited that earlier analysis for this work. PWMs for 14 human transcription factors from JASPAR.2010 which are also present in PAZAR were selected (the ‘PAZAR-J’ dataset) and the performance of the scanning methods was evaluated on these using PAZAR as the gold standard.

TFBS cluster prediction tools chosen were MCast [77], Baycis [78], Cister [79], ClusterBuster [80] and Comet [81] while individual TFBS prediction tools chosen were FIMO [82], Clover [83], Matrix-Scan (part of the RSAT suite) [84], Patser (also part of RSAT) [84] and PossumSearch [85]. Note that Cister, Comet and ClusterBuster all come from the Weng laboratory, with ClusterBuster being their latest software. Consequently this analysis provides an interesting comparison to find out whether their latest software is indeed the best performing.

All tools having variable cutoffs for making predictions were evaluated to ensure the optimum cutoff was chosen by using 10 % steps for all parameters. In all cases, the default settings were found to give the best performance and were used for all future evaluations.

Table 1 shows that FIMO and MCAST are the best performing TFBS prediction tools for individual sites and clusters respectively and FIMO was therefore selected for evaluation of the motif finding methods. (Complete results for individual PWMs are provided in Additional file 2).

**Table 1:** Performance of TFBS prediction methods using JASPAR.2010 PWMs

	*Sn*	*PPV*	*ACC*_*g*_	*FPR*_*s*_
CLUSTER				
Baycis	0.599	0.497	0.545	0.040
Cister	0.635	0.565	0.599	0.037
**MCast**	**0.774**	**0.682**	**0.726**	**0.032**
Comet	0.682	0.589	0.634	0.037
ClusterBuster	0.656	0.580	0.617	0.036
INDIVIDUAL				
Matrix-Scan	0.647	0.579	0.612	0.027
Clover	0.674	0.584	0.627	0.022
**FIMO**	**0.816**	**0.734**	**0.774**	**0.015**
Patser	0.723	0.653	0.687	0.016
PossumSearch	0.708	0.635	0.670	0.019

Average sensitivities (*Sn*), Positive Predictive Value (*PPV*) and geometric accuracy (*ACC*_*g*_) are reported together with the false positive rate using scrambled sequences (*FPR*_*s*_). The best-performing tools, MCast and FIMO are highlighted in bold. Performance was evaluated using the 14 PWMs in the PAZAR-J dataset

### Evaluation of motif discovery methods

We chose to evaluate four methods for motif discovery that have been developed especially for working with large genome-wide datasets and that are open source and well documented: rGADEM [43], HOMER [24], ChIPMunk [42], and MEME-ChIP [44, 45]. For this purpose, TFBS PWMs were derived, using the protocol described above, for the 12 transcription factors in the PAZAR-E dataset.

The tools have parameters that can be adjusted for motif discovery and these were explored for all tools using a 10 % step size. It was found that the defaults produced PWMs that resembled well-established motifs for all tools with the exception of rGADEM where the e-value parameter had to be set to a value of 0.5 rather than the default value of 0.0. The motif discovery tools are also able to generate multiple possible motifs. During the exploration of parameters, it was found that the first PWM generated always best-resembled well-established motifs for the TFBSs used in this work, and consequently only the first PWM was used.

Performance was evaluated by using the FIMO motif scanning tool comparing predictions of TFBS locations with the PAZAR-E data as a gold standard. Table 2 shows that rGADEM has the best performance and MEME-ChIP the worst on all four performance metrics. (Complete results for individual PWMs are provided in Additional file 3 and sequence logos for the first PWM generated for the 12 TFBSs using each of the four motif discovery tools are provided in Additional file 4). We confirmed the finding of Weirauch et al. [56] that ChIPMunk outperforms MEME-ChIP, but showed that rGADEM outperforms both.

**Table 2:** Performance of the different motif discovery tools using FIMO

Motif discovery tool	*Sn*	*PPV*	*ACC*_*g*_	*FPR*_*s*_
ChIPMunk	0.886	0.786	0.834	0.009
HOMER	0.901	0.795	0.846	0.007
MEME-ChIP	0.865	0.771	0.817	0.013
**rGADEM**	**0.933**	**0.839**	**0.884**	**0.002**

Average sensitivities (*Sn*), Positive Predictive Value (*PPV*), geometric accuracy (*ACC*_*g*_) and false positive rate on scrambled sequences (*FPR*_*s*_) are reported. The best-performing tool rGADEM is highlighted in bold. Note that TFBS PWMs were generated only for the 12 transcription factors in the PAZAR-E dataset

The PWMs obtained using the different methods were compared with each other and with those in JASPAR: both the older set derived from SELEX and individual promoter assays (JASPAR.2010) and the newer matrices obtained from ChIP-Seq data (JASPAR.2014). Normalized Euclidean distances between equivalent PWMs were calculated using the TFBSTools package (http://www.bioconductor.org/packages/release/bioc/html/TFBSTools.html) in Bioconductor. Reverse complement matrices were also checked and the minimum distances recorded. Results for each matrix set comparison were averaged across the PWMs used. The normalised Euclidean distance ranges from 0 to 1 where 0 denotes complete identity and 1 denotes complete dissimilarity. Results are shown in Table 3.

**Table 3:** Normalised Euclidean distances between PWMs derived using the different motif discovery tools and PWMs derived from ChIP-Seq, SELEX or individual promoter assays obtained from JASPAR

	JASPAR.2010	JASPAR.2014	rGADEM	HOMER	ChIPMunk	MEME-ChIP
JASPAR.2010	0	—	—	—	—	—
JASPAR.2014	0.393	0	—	—	—	—
rGADEM	0.660	0.404	0	—	—	—
HOMER	0.503	0.234	0.159	0	—	—
ChIPMunk	0.471	0.192	0.263	0.120	0	—
MEME-ChIP	0.404	0.129	0.371	0.203	0.153	0

Note that comparisons between the matrices generated in this work were performed over the 12 TFBS PWMs that were used for performance evaluation (i.e. the PAZAR-E dataset) while the comparisons with JASPAR.2010 and JASPAR.2014 were performed over the 11 PWMs for which binding sites are found in PAZAR and the ENCODE-ChIP-Seq data and which also have PWMs in JASPAR (i.e. the intersection of the PAZAR-E and PAZAR-J datasets)

Comparing the PWMs generated in this work using different motif discovery tools, the best performing method (rGADEM) shows the largest difference in PWMs from the worst performing method (MEME-ChIP). Clearly there are small but significant differences in the PWMs generated by different motif discovery tools. However all the motif discovery methods applied to the ENCODE-ChIP-Seq data show even greater differences from the old JASPAR.2010 PWMs generated using SELEX or individual promoter assays.

### Re-evaluation of PWM scanning tools

Having shown that rGADEM generates better PWMs than other motif-discovery methods, we returned to the evaluation of tools for scanning PWMs against DNA. We repeated this evaluation using PWMs generated from the ENCODE-ChIP-Seq data using rGADEM, and results are shown in Table 4. In general the tools predicting individual sites perform better than those predicting clusters. Because of the more stringent requirements for a true positive in predicting clusters (i.e. *every* predicted site within the cluster must have a 70 % overlap with a true site), it might be expected that the sensitivity for cluster predictors would be lowered, while the specificity would be improved. Indeed the sensitivity of cluster predictors is somewhat lower than the individual site predictors. Since we do not have the true negative count, we cannot calculate specificity, but surprisingly the false positive rate on scrambled sequences (*FPR*_*s*_) for the cluster predictors is larger than that for single site predictors suggesting that the cluster predictors have lower specificity.

**Table 4:** Performance of TFBS prediction methods using the PWMs derived using rGADEM and ENCODE-ChIP-Seq data

	*Sn*	*PPV*	*ACC*_*g*_	*FPR*_*s*_
CLUSTER				
Baycis	0.792	0.687	0.738	0.021
Cister	0.828	0.722	0.773	0.022
MCast	0.907	0.778	0.840	0.013
Comet	0.871	0.759	0.813	0.014
ClusterBuster	0.849	0.739	0.792	0.017
INDIVIDUAL				
Matrix-Scan	0.830	0.717	0.771	0.018
Clover	0.851	0.736	0.791	0.015
**FIMO**	**0.933**	**0.839**	**0.884**	**0.002**
Patser	0.887	0.774	0.828	0.008
PossumSearch	0.875	0.758	0.814	0.010

Average sensitivities (*Sn*), Positive Predictive Value (*PPV*) and accuracy (*ACC*_*g*_) are reported together with the false positive rate using scrambled sequences (*FPR*_*s*_). Performance was evaluated across the 12 PWMs that could be derived from the ENCODE-ChIP-Seq data using rGADEM that have validated TFBSs in PAZAR (the PAZAR-E dataset). The best performing tools, MCast and FIMO are highlighted in bold

Using the JASPAR.2010 data, we had identified FIMO as the best tool for identifying individual TFBSs and MCast as the best cluster-based tool. Table 4 shows that these two tools still perform best using the PWMs derived here using rGADEM and ENCODE-ChIP-Seq data. (Complete results for individual PWMs are provided in Additional file 5). Indeed the overall ranking of all the tools remains the same:

*MCast* >*Comet* >*ClusterBuster* >*Cister* >*Baycis*for cluster predictors and

*FIMO* >*Patser* >*PossumSearch* >*Clover* >*Matrix-Scan*for individual predictors.

Cister, Comet and ClusterBuster all come from the same laboratory (published in 2001, 2002 and 2003 respectively). These results suggest that Comet from 2002 outperforms ClusterBuster from 2003, but both have made progress over their initial 2001 software. However MCast significantly outperforms all three methods.

## Conclusions

As a comprehensive set of experimentally-characterized precise transcription factor binding sites is not available, having good reliable prediction methods is very important. While some experimental methods of identifying TFBSs are relatively accurate, identifying regions of around 10–20 b.p., methods such as ChIP-Chip, and more importantly the ‘gold standard’ ChIP-Seq method, identify DNA regions of 100–1000 b.p. which is much larger than the TFBS itself (typically 9–15 b.p.). Consequently, when these experimental methods are employed for identifying TFBSs, it is necessary to use a prediction tool to identify the TFBS within the much wider region. While the need for identifying TFBSs as an adjunct to gene prediction in the human genome has diminished, it is now much more important in order to have a full understanding of the regulation of gene expression and to be able to consider the potential phenotypic effects of mutations occurring in a TFBS.

### Motif discovery

None of the ENCODE-ChIP-Seq data used to derive the PWMs for evaluating motif discovery tools overlapped the sequences obtained from genes present in PAZAR and consequently we know there is no overlap between the training and test sets. Table 2 clearly shows that PWMs derived using rGADEM outperform those derived using other motif discovery methods.

### Alternative sources of binding data

The analysis here has focused on the use of data from ChIP-Seq experiments which, as described in the introduction, have largely superseded the earlier ChIP-chip approach; both of these are in vivo approaches. Another relatively new approach is the in vitro SELEX-seq [11] approach. To investigate whether SELEX-seq would be a useful addition to ChIP-Seq data, we used rGADEM with SELEX-seq data to derive a PWM for NF- *κ*B, the only transcription factor for which SELEX-seq, ENCODE-ChIP-Seq data and PAZAR data are available.

The performance of the SELEX-seq derived PWM (*Sn*=0.913, *PPV*=0.810, *ACC*_*g*_=0.860, *FPR*_*s*_=0.004) is less than its counterpart derived from the ENCODE-ChIP-Seq data (*Sn*=0.937, *PPV*=0.831, *ACC*_*g*_=0.882, *FPR*_*s*_=0.002). However no firm conclusions can be drawn on the performance of SELEX-seq data in general on the basis of a single transcription factor.

Another recently developed technology is ChIP-exo [17]. Unfortunately no data are available from ChIP-exo for TFBSs that are present in the PAZAR gold standard dataset and consequently we cannot evaluate the performance of PWMs derived from these data.

### Scanning tools

An inherent problem with TFBS prediction is their short and degenerate nature. The non-redundant vertebrate TFBS PWMs in JASPAR.2014 range from 5 b.p. (Pax4) to 30 b.p. (Prrx2), but with the majority being 9–15 b.p. (mean =12.2, *σ*=3.7). A naïve scanning of PWMs against a DNA sequence can therefore result in a high false positive rate. It is therefore essential to optimize the methods used to scan a PWM against a DNA sequence in order to minimize the false positive rate.

We have evaluated a set of transcription factor binding site prediction tools that could be downloaded and installed locally, identifying FIMO and MCAST as the best-performing tools for identifying individual TFBSs and clusters of TFBSs respectively. While it is possible that there is some inter-relationship between the choice of motif discovery method and the tool used to search those motifs against a DNA sequence, this seems unlikely to be significant. The ranking of tool performance was the same when used with the JASPAR.2010 PWMs (generated using MEME-based tools) and the PWMs generated in this work using rGADEM. Similarly, using FIMO (part of the MEME suite) as a search tool, PWMs generated using MEME-ChIP do not perform as well as PWMs generated using rGADEM (Table 2).

### Alternatives to PWMs

Position Weight Matrices (PWMs) are the most widely used TFBS models, but are limited by the assumption of the model that positions within a binding site are independent, something which is not true in all cases [56]. There have therefore been several attempts to develop more complex alternatives to the PWM model that take into account nucleotide interdependencies [6, 18, 25]. Some examples include pair-correlation models [86], trees [87], non-parametric models [88], feature-based models [89], Markov chain optimization [90], maximal dependence decomposition [91], Hidden Markov Models [92], transcription factor flexible models [93] and Dinucleotide PWMs [94].

However it has been observed that classical PWM models tend to perform at least as well as more complex models [18] and that more complex models tend to be prone to learning noise. Consequently, it has been suggested that the PWM model may be the state of the art and that focus should be placed on optimizing the PWM model rather than developing more complex models [95].

While PWMs are not outperformed by more complex models for the majority of transcription factors, for a small number of individual transcription factors it has been found that more complex models do result in better performance [56]. For example, more complex models perform better for transcription factors AP-2A and REST, but not for HNF4A [94]. Thus, in future, it may be worth evaluating both PWMs and more complex models and selecting an appropriate model for each individual transcription factor.

### Summary

While TFBS predictors which identify individual sites outperform those that identify clusters, the choice of the type of prediction tool depends on the context in which it is to be used. The evaluation used in this study was performed in the context of known TFBSs associated with genes. Consequently, if prior knowledge is available about the DNA sequence being scanned (i.e. the DNA sequence is that of a known protein coding gene) then using a predictor of individual TFBSs is probably a sensible strategy. When analyzing a stretch of DNA with no prior knowledge about the presence of a gene, it would be better to use a prediction tool that identifies clusters of TFBSs since the chance of a random match is much reduced [58, 96].

In conclusion, we have analyzed motif discovery tools for generating PWMs from ChIP-Seq data using experimentally-validated precise TFBSs from PAZAR as a gold standard. We found that rGADEM out-performed other tools. We then evaluated a number of tools for scanning PWMs against DNA, both for identifying individual TFBSs and clusters of TFBSs. We found that FIMO and MCAST performed best respectively. We also found that there appears to be no dependence between the tool used for motif discovery and the tool used for motif scanning — in other words, using (for example) a motif scanning tool from the MEME suite does not perform better when using PWMs generated using a motif discovery tool from the MEME suite than when using an unrelated motif discovery tool.

## Additional files

Additional file 1 The PAZAR Reference Dataset. Entries from PAZAR, with the numbers of each TFBS that they contain. The spreadsheet also indicates whether the TFBSs are found in JASPAR and/or the ENCODE-ChIP-Seq data. (XLS 54 kb)

Additional file 2 Evaluation of search tools using JASPAR.2010 PWMs. Sensitivity, Positive predictive value, Geometric accuracy and False positive rate for the 14 TFBSs that are found in the PAZAR-J dataset. Separate sheets are provided for each of the search tools. (XLS 31.5 kb)

Additional file 3 Motif Discovery Tool Performance. Complete results for individual PWMs generated using different motif discovery tools and scanned against PAZAR-E data using the FIMO motif scanning tool. (XLS 14 kb)

Additional file 4 Sequence Logos. Sequence logos for the first PWM generated for the 12 TFBSs using each of the four motif discovery tools. (PDF 29.5 kb)

Additional file 5 Evaluation of search tools using PWMs generated from the ENCODE-ChIP-Seq data using rGADEM. Sensitivity, Positive predictive value, Geometric accuracy and False positive rate for the 12 TFBSs that are found in the PAZAR-E dataset. Separate sheets are provided for each of the search tools. (XLS 29.5 kb)

## Abbreviations

ChIP: Chromatin immunoprecipitation
EMSA: Electro-mobility shift assay
PWM: Position weight matrix
SNV: Single nucleotide variant
SELEX: Systematic evolution of ligands by exponential enrichment
TFBS: Transcription factor binding site
