# Supplementary material for: Evaluating tools for transcription factor binding site prediction
Source: BMC Bioinformatics. 2016 Nov 2;17:547. doi: 10.1186/s12859-016-1298-9 (PMC6889335; doi:10.1186/s12859-016-1298-9)
Supplement: Supplementary file 4 — Sequence Logos. Sequence logos for the first PWM generated for the 12 TFBSs using each of the four motif discovery tools. (PDF 29.5 kb) [file 12859_2016_1298_MOESM4_ESM.pdf]

Evaluating tools for transcription factor binding site prediction

Sequence Logos

N. Jayaram, D. Usvyat and A.C.R. Martin

Ctcf

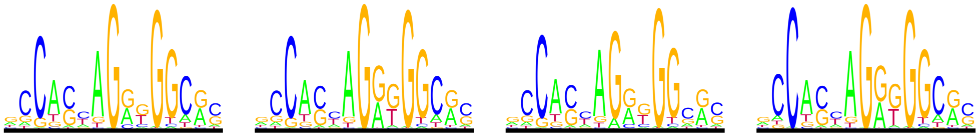

Chipmunk Homer MEME-Chip rGADEM

E2F1

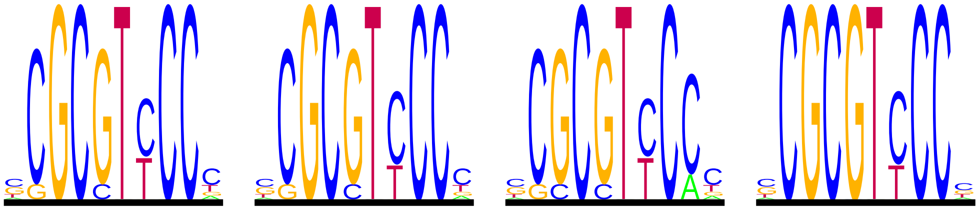

Chipmunk Homer MEME-Chip rGADEM

ELK4

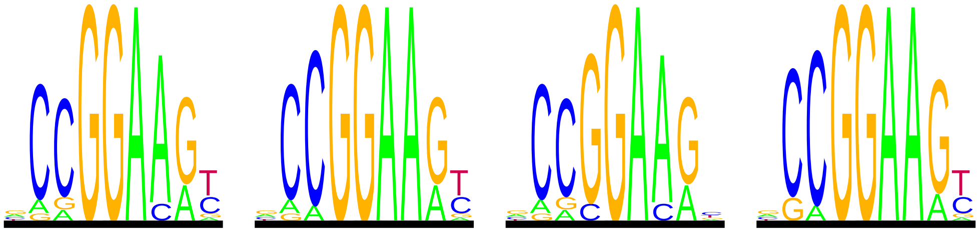

Chipmunk Homer MEME-Chip rGADEM

GATA2

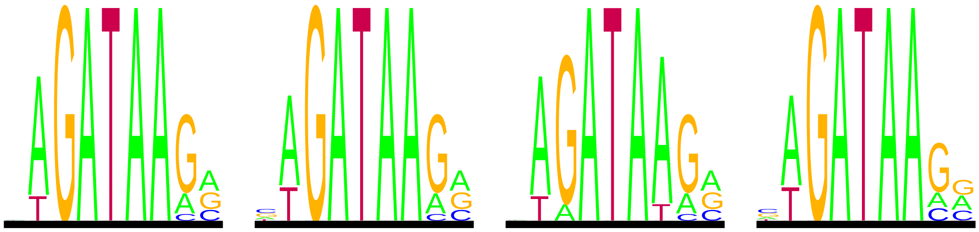

Chipmunk Homer MEME-Chip rGADEM

GATA3

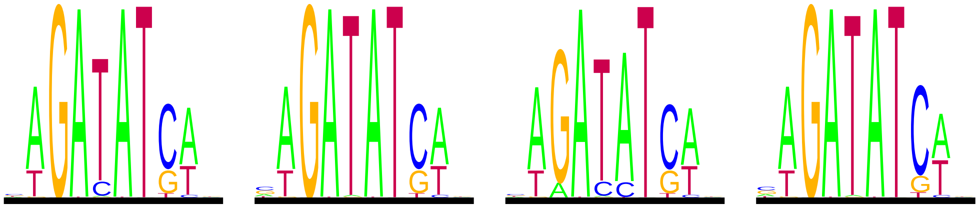

Chipmunk Homer MEME-Chip rGADEM

IRF1

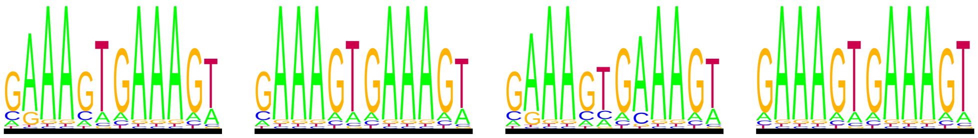

Chipmunk Homer MEME-Chip rGADEM

MAX

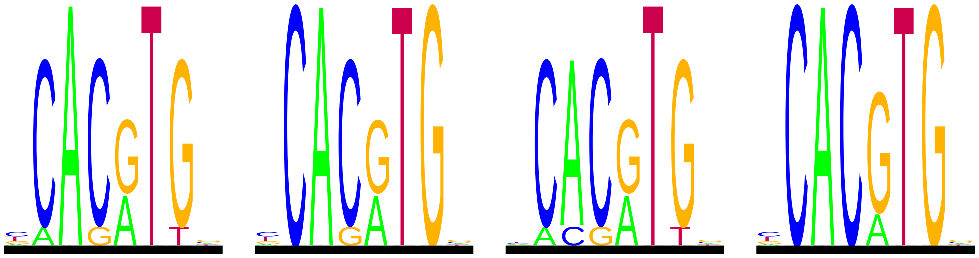

Chipmunk Homer MEME-Chip rGADEM

NFKB

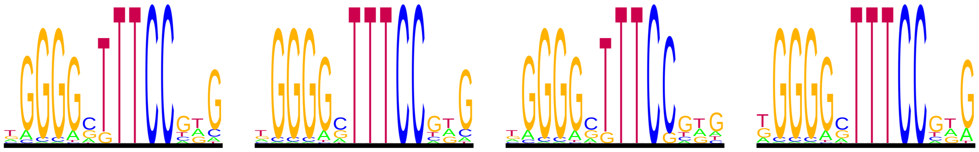

Chipmunk Homer MEME-Chip rGADEM

NF-YA

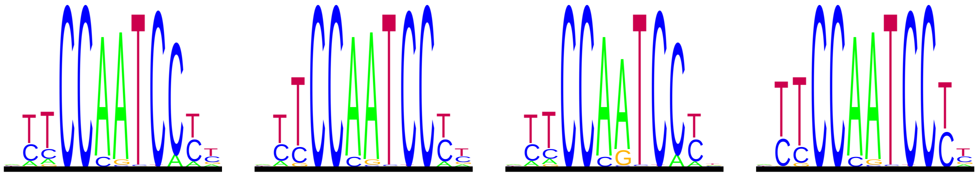

Chipmunk Homer MEME-Chip rGADEM

STAT1

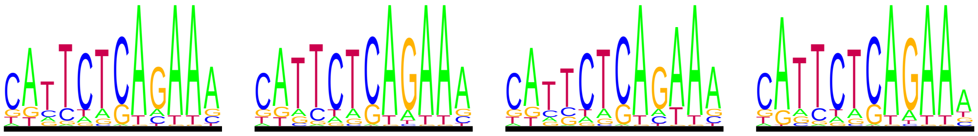

Chipmunk Homer MEME-Chip rGADEM

TAL1

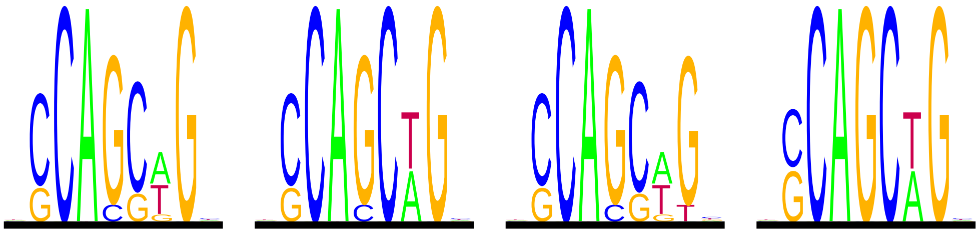

Chipmunk Homer MEME-Chip rGADEM

YY1

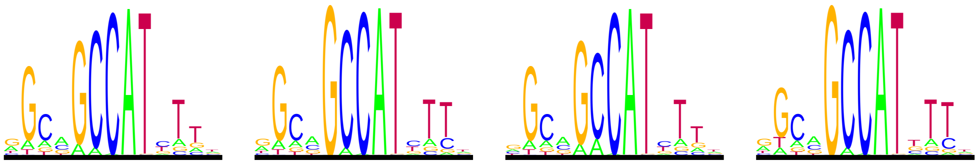

Chipmunk Homer MEME-Chip rGADEM
